# The complete mitochondrial genome of *Oberthueria lunwan* (Lepidoptera: Oberthuerinae)

**DOI:** 10.1080/23802359.2017.1413292

**Published:** 2017-12-13

**Authors:** Ming-Wei Liao, Xing-Shi Gu, Jin Xue, Xing Wang

**Affiliations:** a College of Plant Protection, Hunan Agricultural University, Changsha, China;; b Hunan Provincial Key Laboratory for Biology and Control of Plant Diseases and Insect Pests, Hunan Agricultural University, Changsha, China

**Keywords:** Mitochondrial genome, Oberthuerinae, *Oberthueria lunwan*, evolutionary position

## Abstract

In this study, the complete mitochondrial genome (mitogenome) of *Oberthueria lunwan* Zolotuhin & Wang, 2013 (Lepidoptera: Oberthuerinae) is reported for the first time. The entire mitogenome is a circular DNA molecule of 15,673 bp in length (GenBank accession number: MF100143), consisting of 13 protein-coding genes (PCGs), 22 transfer RNAs (tRNAs) genes, two ribosomal RNAs (rRNAs) genes, and a control region (A + T-rich region). The phylogenetic trees are based on 13 PCGs amino acid sequences of 31 related lepidopteran species in which mitogenome sequences were constructed. The Oberthuerinae consisting of *O. lunwan*+*Andraca theae* was strongly supported as a monophyletic clade by the posterior probability of 1.00 and the bootstrap value of 100%, but the relationships among Oberthuerinae, Bombycidae, Satruniidae, and Sphingidae are need to be further confirmed.

The *Oberthueria* species are difficult for distinguishing from each other in general appearance (Zolotuhin and Wang 2013) with their complete mitochondrial genome (mitogenome) unreported. *Oberthueria lunwan* Zolotuhin and Wang, 2013 (Lepidoptera: Oberthuerinae) was previously recorded just from mainland China (Yunnan) and northeastern Myanmar with the larval host unknown (Wang et al. 2015). Here, the adults of this species were collected from Shennonggu National Forest Park (26°18′ 00″ N, 113°56′ 30″ E, 900 m at attitude), Yanling County, Hunan Province, Centre China on 12 September 2015. The genetic characters of *O. lunwan* are very helpful for understanding its phylogenetic relationships in the superfamily Bombycoidea and identifying the *Oberthueria* species correctly.

The mitogenome DNA was extracted from the adult’s thorax muscles and purified by using Wizard Genomic DNA Purification Kit (Promega, Beijing, China). All the adult specimens and the genomic DNA were deposited in Insect Museum of Hunan Agricultural University. Primers reported by Gu et al. (2016) were used to amplify the complete mitogenome. The fragments were proof-read by using the software of Geneious version 8.1.2 (Kearse et al. 2012). The gene annotation was accomplished with MITOS (http://mitos.bioinf.uni-leipzaig.de) (Bernt et al. 2013). Filtrating the nonconservative parts was done using Gblocks 0.91b (Castresana 2000). The phylogenetic trees of the Bombycoidea with Lasiocampidae species as outgroups were established by using MrBayes 3.1.2 (Ronquist et al. 2012) with running for 5,000,000 generations and using raxmlGUI 1.5 program (https://sourceforge.net/projects/raxmlgui/) (Silvestro and Michalak 2012) with 1000 replications.

The complete mitogenome of *O. lunwan* is a circular DNA molecule of 15,673 bp in length, consisting of 13 protein-coding genes (PCGs), 22 transfer RNAs (tRNAs) genes, two ribosomal RNAs (rRNAs) genes, and a control region (A + T-rich region). The mitogenome contains A (40.4%), T (39.3%), C (12.4%), and G (7.9%), which showed a high A + T bias. All of the PCGs started with ATN as the start codon except for *cox1*, which started with CGA. In addition, 11 of the 13 PCGs ended with TAA, whereas *cox1* and *cox2* ended with a single T. The A + T-rich region of the full-length 551 bp, located between *rrnS* and *trnM* gene, had 93.28% A + T content. The conserved region included an ‘ATAGT’ structure followed by poly-T. There were two microsatellites, ‘(TA)_6_’ and ‘(TA)_8_’, which were located at 86 bp and 202 bp upstream of *rrnS*, respectively.

Based on the amino acid sequences of the 13 PCGs from 31 related lepidopteran species (Wang et al. 2015; Gu et al. 2016; Liu et al. 2017; Wu et al. 2017; Zhao et al. 2017), the phylogenetic trees were established with the 24 Bombycoidea species as ingroups and 7 Lasiocampidae species as outgroups. The maximum likelihood (ML) and Bayesian inference (BI) trees showed the same topological structures (Figure 1). The Oberthuerinae consisting of the species *Andraca theae* and the *O. lunwan* formed a monophyletic clade supported by the bootstrap value of 100% and the posterior probability of 1.00. The phylogenetic relationships among Sphingidae, Bombycidae, Satruniidae, Oberthuerinae in Bombycoidea were uncertain with further studies necessary.

**Figure 1.**
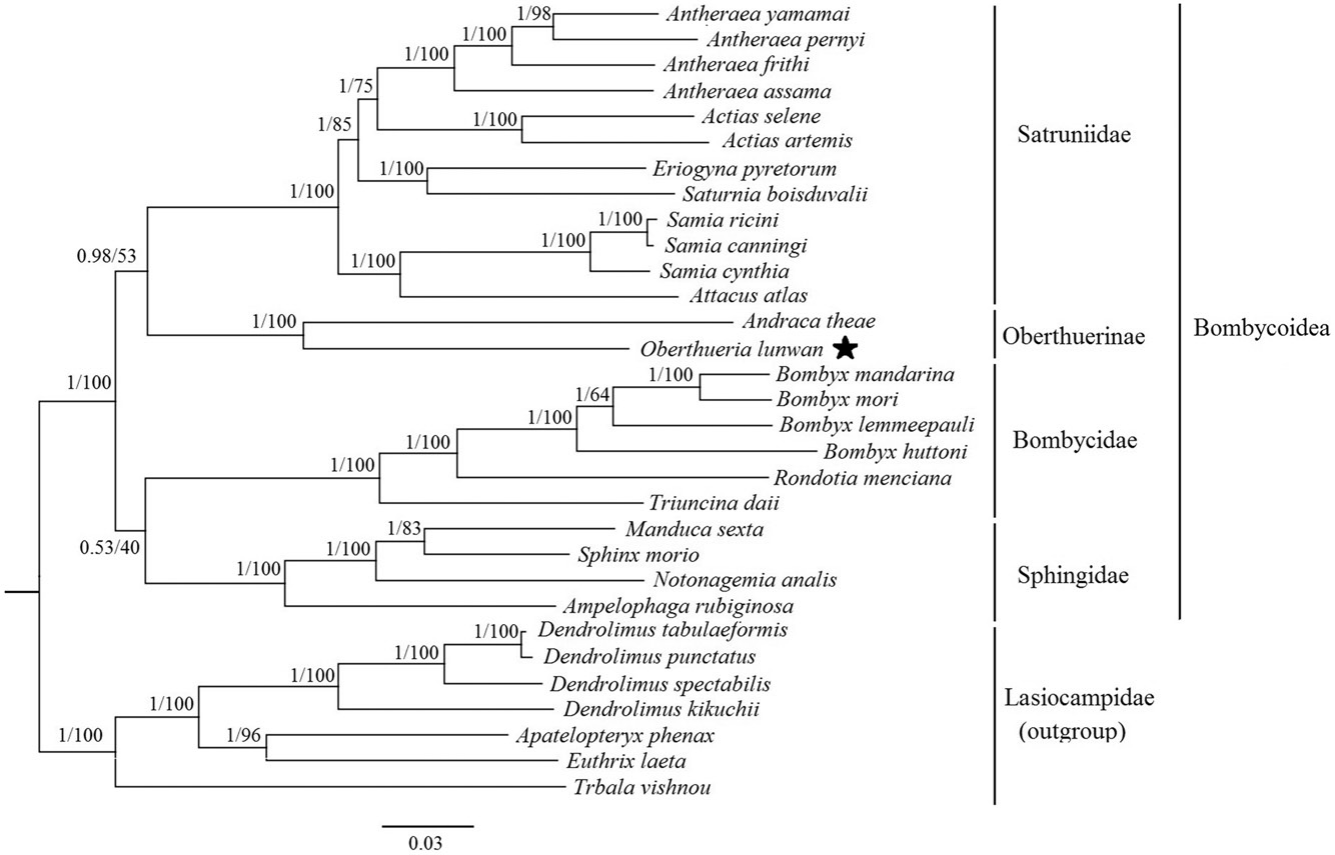
Bayesian inference and maximum likelihood phylogram constructed using 13 PCGs of mitogenomes with partitioned models. Numbers above each node indicate the ML bootstrap support values and the BI posterior probability. All the species’ accession numbers in this study are listed as below: *Actias artemis* KF_927042, *Actias selene* NC_018133, *Ampelophaga rubiginosa* NC_035431, *Andraca theae* KX365419, *Antheraea assama* NC_030270, *Antheraea frithi* NC_027071, *Antheraea pernyi* NC_004622, *Antheraea yamamai* NC_012739, *Apatelopteryx phenax* KJ508055, *Attacus atlas* NC_021770, *Bombyx huttoni* NC_026518, *Bombyx lemeepauli* KY620670, *Bombyx mandarina* NC_003395, *Bombyx mori* NC_002355, *Dendrolimus kikuchii* MF100138, *Dendrolimus punctatus* NC_027156, *Dendrolimus spectabilis* NC_025763, *Dendrolimus tabulaeformis* NC_027156, *Eriogyna pyretorum* NC_012727, *Euthrix laeta* NC_031507, *Manduca sexta* NC_010266, *Oberthueria lunwan* MF100143, *Rondotia menciana* NC_021962, *Samia canningi* NC_024270, *Samia cynthia* KC812618, *Samia ricini* NC_017869, *Saturnia boisduvalii* NC_010613, *Sphinx morio* NC_02078, *Notonagemia analis* KU934302, *Trabala vishnou* KU884483, *Triuncina daii* KY091643.
